# Unfolding the Role of Large Heat Shock Proteins: New Insights and Therapeutic Implications

**DOI:** 10.3389/fimmu.2016.00075

**Published:** 2016-03-01

**Authors:** Daming Zuo, John Subjeck, Xiang-Yang Wang

**Affiliations:** ^1^Department of Immunology, Southern Medical University, Guangzhou, China; ^2^State Key Laboratory of Organ Failure Research, Department of Infectious Diseases, Nanfang Hospital, Southern Medical University, Guangzhou, China; ^3^Guangdong Provincial Key Laboratory of Viral Hepatitis Research, Department of Infectious Diseases, Nanfang Hospital, Southern Medical University, Guangzhou, China; ^4^Department of Cellular Stress Biology, Roswell Park Cancer Institute, Buffalo, NY, USA; ^5^Department of Human and Molecular Genetics, Virginia Commonwealth University, Richmond, VA, USA; ^6^VCU Massey Cancer Center, Virginia Commonwealth University, Richmond, VA, USA; ^7^VCU Institute of Molecular Medicine, Virginia Commonwealth University, Richmond, VA, USA

**Keywords:** heat shock protein 110, glucose-regulated protein 170, cytoprotection, innate immunity, antigen cross-presentation, inflammatory disease

## Abstract

Heat shock proteins (HSPs) of eukaryotes are evolutionarily conserved molecules present in all the major intracellular organelles. They mainly function as molecular chaperones and participate in maintenance of protein homeostasis in physiological state and under stressful conditions. Despite their relative abundance, the large HSPs, i.e., Hsp110 and glucose-regulated protein 170 (Grp170), have received less attention compared to other conventional HSPs. These proteins are distantly related to the Hsp70 and belong to Hsp70 superfamily. Increased sizes of Hsp110 and Grp170, due to the presence of a loop structure, result in their exceptional capability in binding to polypeptide substrates or non-protein ligands, such as pathogen-associated molecules. These interactions that occur in the extracellular environment during tissue injury or microbial infection may lead to amplification of an immune response engaging both innate and adaptive immune components. Here, we review the current advances in understanding these large HSPs as molecular chaperones in proteostasis control and immune modulation as well as their therapeutic implications in treatment of cancer and neurodegeneration. Given their unique immunoregulatory activities, we also discuss the emerging evidence of their potential involvement in inflammatory and immune-related diseases.

## Introduction

Heat shock proteins (HSPs) are constitutively expressed in all living organisms. The HSP nomenclature, originated from the observation of their induction by thermal stress, may also be used to describe other stress-inducible proteins, such as glucose-regulated proteins or GRPs (1–3). In contrast to cytoplasm-resident HSPs, GRPs mainly reside in the endoplasmic reticulum (ER). The mammalian HSPs are classified into several families of proteins based on their molecular size. Hsp110 and Grp170 are high molecular weight HSPs, which have been less studied compared to other conventional HSPs, e.g., Hsp70 and Hsp90 (4, 5). The transcription and subsequent protein expression of Hsp110 or Grp170 are stimulated by cytotoxic or proteotoxic stresses, such as heat shock, oxidative stress, chemical agents, hypoxia, viral infections, and inflammation. In such situations, these large HSPs work in concert with other chaperone molecules (e.g., Hsp70, Grp78) to restore protein folding and cellular function to counter proteotoxic stresses and promote cell survival (6–8). This intrinsic chaperoning feature of the large HSPs has significant implications in several diseases including neurodegeneration and cancer.

Given the superior protein-holding activity of these large HSPs and their unique ability to interact with the immune system, Hsp110 and Grp170 have been used to induce antigen/tumor-reactive immune response for cancer eradication (5, 9, 10). These large HSPs can broadly chaperone antigens for presentation to T lymphocytes and amplify inflammatory signals in the extracellular environment, suggesting a potential endogenous immunostimulatory effects of these large HSPs during tissue stress or injury (e.g., microbial infection). Although the mechanism of their intracellular or extracellular action still needs to be elucidated, emerging evidence indicates that these large HSPs may be involved in regulation of an inflammatory response and bridging innate and adaptive immunity, which may contribute to the pathogenesis of inflammatory and immune-related diseases. Here, we review the accumulating evidence of multifaceted functions of these large HSPs under physiological or pathological conditions and discuss the potential applications that exploit the distinct activity of the large HSPs to achieve therapeutic benefits.

## The Large HSPs: Distribution, Induction, and Regulation

Hsp110, also referred to as Hsp105, is one of the major eukaryotic HSPs and a member of conservative Hsp70 superfamily (4, 5). The studies of Hsp110 have been largely ignored until the cloning of its cDNA in the early 1990s (11–13). Hsp110 has been well characterized in mammals (i.e., human, mouse, and hamster) (11, 14–19). There appears to be two forms of Hsp110, 105α, and 105β (43 fewer amino acids than 105α) (14). Hsp105α is expressed constitutively in the cytoplasm of cells and can be further induced by heat shock and other stressors, whereas Hsp105β, an alternatively spliced form of Hsp105α specifically localized in the nucleus, is strictly heat-inducible (20). However, the differential roles potentially played by these two versions of Hsp110 remain not well studied given their distinct compartmentalization. As one of the major HSPs, Hsp110 is easily detectable in most mammalian cell lines and tissues (11, 21). Hsp110 is constitutively expressed in the various tissues, e.g., brain, liver, ovary, spleen, heart, lung, small intestine, and muscles, but most abundant in the brain (13, 14, 22). Interestingly, mammalian cerebellum expresses little Hsp110 compared to other brain regions (13). It is not clear if the lack of Hsp110 expression contributes to the high sensitivity of the cerebellum to heat stroke or alcohol-associated toxicity (23, 24).

Hsp110 along with other HSPs could be induced in a specific set of stress conditions including hyperthermia, ethanol, oxidative reagents, recovery from anoxia (i.e., reperfusion injury), and inflammation (25, 26). Studies of heat shock factor (HSF) knockout mice indicated that the transcription factor HSF1 is required for induction of Hsp110, Hsp70, and Hsp25/27, by thermal stress (27). Exposure of BALB/c mice to fever-range whole body hyperthermia (39.5–40°C for 6 h) enhanced Hsp110 expression in the lung, lymph nodes, and thymus (28). However, thermal stress did not appear to influence the Hsp110 levels in the rat nervous system (29, 30). Peptide mapping analysis and use of various deletion or substitution mutants revealed that Hsp110 is phosphorylated at Serine (509) in the β-sheet domain by casein kinase II (31). An early study reported that Hsp110 suppressed 70-kDa heat-shock cognate protein (Hsc70)-mediated protein folding, while the phosphorylation of Hsp110 diminished its inhibitory activity *in vitro* (32). Intriguingly, phosphorylated Hsp110 is especially prominent in the brain compared to other tissues of mice or rats, suggesting that the phosphorylation of Hsp110 may be physiologically significant (33).

Like other HSPs reported to affect the morphologic development of cells and organisms (34), Hsp110 is also important for reproductive and embryonic development. Fujita and coworkers cloned two Hsp110 cognate cDNAs from testis, designated apg-1 and apg-2 (17, 35, 36). Apg-1 is developmentally expressed in human testicular germ cells and sperm, suggesting its potential role in spermatogenesis and fertilization (17, 35, 37). Injection of antisense oligodeoxynucleotides targeting Hsp110 into the rat uterine horn on day 3 of pregnancy substantially reduced the number of the implanted embryos (38), implicating a physiological role for Hsp110 in regulation of reproduction. Indeed, Hsp110 displayed a differential expression pattern associated with the development of palate in mouse embryo (39). A recent study showed that Hsp110 plays an essential role in embryonic development of mouse hindlimbs (40). Strikingly, the integrity of Hsp110 in β-catenin degradation complex is required for Wnt signaling pathway, which can lead to embryonic defects upon abnormal activation (41, 42).

Grp170, also known as oxygen-regulated protein 150 (Orp150) or hypoxia-upregulated protein 1 (HYOU1), was first described as a 170 kDa molecule inducible by glucose starvation (43). Hypoxia was soon found as a physiological inducer of Grp170 (44–48). Cloning and sequence analysis of Grp170 indicated that Grp170 represented a new stress protein family (12). Although they have a somewhat greater degree of sequence similarity to the Hsp110 family sequences than to members of the Hsp70 family, the Grp170s of mammals are essentially as diverged from the Hsp110 family of proteins as they are from the Hsp70s (4, 12, 49, 50).

Similar to other ER-resident GRPs (e.g., Grp78, Grp94/gp96), Grp170 possesses a C-terminal ER retention sequence, and therefore localizes in the ER after protein synthesis. Grp170 is inducible by glucose starvation, chronic anoxia/hypoxia, calcium depletion, low pH, a variety of reducing conditions, stress-induced activation of the hypothalamic–pituitary–adrenal (HPA) axis, and viral infection that perturb the ER function (44, 51, 52), suggesting that Grp170 participates in quality control of protein folding in the ER. Grp170 is co-regulated with Grp78 and Grp94/gp96 by the unfolded protein response (UPR) (53). Transcription of these UPR-inducible genes is regulated by ER stress–response elements (ERSE) and key transcription factors such as the X-box DNA binding protein 1 (XBP-1) and ATF6 (54–57). In addition, Grp170 was also found to localize in the mitochondria and could be upregulated by CHOP/GADD153 in response to mitochondrial and ER stress (58).

Although HSPs are generally considered to be intracellular proteins, they can be mobilized to the plasma membrane or released into the extracellular environment (59). HSPs do not seem to be secreted from cells *via* the classical pathway since they do not possess a signal peptide. Nonetheless, HSPs may be released from cells through either a passive (e.g., cell injury) or an active (translocation to the membrane for secretion) process. Hightower et al. first reported that rat embryo cells upon heat-shock rapidly released Hsp70 and Hsp110 (60). The release of Hsp110 from human intestinal epithelial cells (IECs) was also observed in the physiological process of epithelial renewal, and this release was further increased upon pathological cell death (61). It was shown that Hsp110 preferentially associated with and stabilized misfolded protein in both the ER and cell periphery (62), suggesting Hsp110-based chaperoning activity may be involved in protein homeostasis at different locations. Recently, presence of Hsp110 on the plasma membrane of human B-cell non-Hodgkin lymphoma (B-NHL) cell lines was reported to correlate to the aggressiveness of lymphoma (63). Interestingly, Grp170 was identified on the surface of human sperm, implicating its potential physiological role in reproduction and fertilization (64). Additionally, Grp170 was found to be released from human cancer cells to the circulating system in patients with a range of neoplasms (65). Our study showed that both Hsp110 and Grp170 can gain access to the extracellular space upon cell necrosis and exhibit immunoregulatory activity (66). Although the biological significance of these surface or extracellular Hsp110 and Grp170 remain to be determined, these large HSPs are likely to exhibit multifaceted effects at cellular sites under physical and pathological conditions.

The constitutively expressed Hsc70, also known as HSPA8, and stress-inducible Hsp70 are central players in proteostasis control, including *de novo* folding, refolding of stress-denatured proteins, oligomeric assembly, protein trafficking, and proteolytic degradation (67). It has long been recognized that Hsp110 exists in multi-protein complexes containing Hsc70/Hsp70 (68–70) and Grp170 associates with other major GRPs (e.g., Grp78, Grp94/gp96) in the ER (51, 71), suggesting coordinated activities of chaperones in the maintenance of protein homeostasis. Our studies demonstrate that Hsp110 and Grp170 are highly effective in inhibiting the aggregation of heat-denatured protein substrates (50, 68, 72–74). The genetic evidence also came from the observation that the Hsp110 deficiency impaired the refolding of heat-denatured luciferase in mammalian cells (75). Not surprisingly, Hsp110, Hsp70, and Hsp40 constitute a disaggregase machinery with capacity to efficiently disaggregate and refold aggregates of chemically or thermally denatured protein (76, 77), further highlighting a role of large HSP in molecular chaperoning and protein homeostasis. Hsp110 (78–81) and Grp170 (82–84) were recently proposed to serve as a nucleotide exchange factor (NEF) for Hsc70/Hsp70 and Grp78, respectively, during ATP hydrolysis of chaperoning cycle. Given the high efficiency of Hsp110 or Grp170 in protein holding, and their differential binding preferences for peptide residues compared to Hsc70/Hsp70 (85), we believe that Hsp110 or Grp170 do not function solely as a co-chaperone or a NEF in cellular functions.

## Large HSP-Mediated Cytoprotection and Tissue Homeostasis

The intrinsic chaperoning property of large HSPs primarily contributes to their cytoprotective functions under stress conditions. Other mechanisms underlying their pro-survival activity may involve HSP-mediated suppression of the intracellular apoptotic pathways (86). We have demonstrated that both Hsp110 and Grp170 are significantly more efficient than Hsc70/Hsp70 in stabilizing and preventing irreversible aggregation of heat-damaged protein (50, 68, 72–74), which may be attributed to the large size of these two chaperones due to the substantial expansion of their C-terminuses (4, 5). The overexpression of Hsp110 in Rat-1 and HeLa cells conferred partial thermotolerance and promoted cell survival to a severe heat shock (68). Eroglu et al. recently reported that Hsp110-deficient mice exhibit more serious brain damage and edema in controlled cortical impact model for traumatic brain injury (TBI) compared to wild-type counterparts (22). Enhancing the expression of the Hsp110 by celastrol treatment effectively reduced injury at the impact site (22), suggesting a potential benefit of using drugs to induce Hsp110 for reducing the pathological effects of TBI. Similarly, Grp170 was highly upregulated in astrocytes undergoing hypoxic stress (47), suggesting a cellular protective role of Grp170. Grp170 overexpression in cultured neurons increased their resistance to hypoxic/ischemia stress, whereas astrocytes with reduced Grp170 expression became vulnerable (87). Upregulation of Grp170 also effectively ameliorated hepatic ER stress and hypercholesterolemia-related liver damage (88).

The accumulation of misfolded or aggregated protein that is non-functional and cytotoxic has been implicated in the pathogenesis of neurodegenerative diseases (89). While it has been well established that conventional HSPs (e.g., Hsp70 or Hsp90) suppress the neurotoxicity associated with protein misfolding both *in vitro* and *in vivo*, emerging evidence indicates an essential neuroprotective effect of large HSPs. Mutant human Cu/Zn superoxide dismutase 1 (SOD1) is associated with motor neuron toxicity and cell death in an inherited form of amyotrophic lateral sclerosis (ALS; Lou Gehrig disease). Song et al. showed that Hsp110 could prevent the aggregation of misfolded SOD1 species, resulting in abrogation of the neuron toxicity by rescuing the transport defect in transgenic mice and in axoplasm isolated from squid giant axons (90). A growing body of evidence suggests that soluble alpha-amyloid precursor protein (sAPPalpha), a cleavage product of APP, has neurotrophic and neuroprotective properties in Alzheimer disease (91). A recent study reported that Grp170 induced by sAPPalpha is part of this neuroprotective response (92). Erogu et al. provided first *in vivo* genetic evidence supporting the role of Hsp110 in pathogenesis of Alzheimer’s disease (93). It was shown that Hsp110-deficient mice exhibited accumulation of hyperphosphorylated-tau and neurodegeneration. Crossing Hsp110-deficient mice with animal strain overexpressing mutant APP resulted in selective appearance of insoluble amyloid β42. Analysis of brain tissues from patients with Alzheimer’s disease showed the expression of Hsp110 in close proximity to Aβ plaques. Together, these results underscore an essential role for Hsp110 in maintaining the proper folding environment that is required for phosphorylation/dephosphorylation of tau and APP processing *in vivo*.

High expression of HSPs that has been well documented in a wide range of human cancer cells supports their crucial role in proliferation and survival of tumor cells. Moreover, the levels of HSPs have been proposed to be useful prognostic biomarkers for tumorigenesis in some cancers (94, 95). Hsp110 is one of the most highly upregulated proteins in a variety of human cancers (96) and can suppress cancer cell apoptosis by preventing the release of cytochrome *c* from the mitochondria (97–99). The small interfering RNA-mediated downregulation of Hsp110-sensitized human cancer cells to apoptotic induction (99). Yu et al. recently provided new insight into the molecular mechanism of Hsp110 overexpression in tumorigenesis (41). Hsp110 expression was correlated to upregulation of β-catenin and transcription of Wnt target genes in many cancers, including colorectal cancer and breast cancer. Knockdown of Hsp110 disrupted the integration of PP2A into the β-catenin degradation complex, resulting in degradation of β-catenin and inhibition of proliferation of colon cancer cell lines that harbor adenomatous polyposis coli (APC) mutation (41). Given that the majority of colon cancer patients have APC mutations and active Wnt/β-catenin signaling, these findings establish Hsp110 as a prognostic biomarker and as a potential therapeutic target for the treatment of colon cancer (100, 101).

## Large HSPs in Innate Immunity

Several reports have documented the effect of endotoxin free large HSPs (i.e., Hsp110 and Grp170) in inducing co-stimulatory signals and cytokine responses in innate immune cells, such as dendritic cells (DCs) (102, 103). Tumor cells engineered to produce a secretable form of Grp170-stimulated DCs to produce the pro-inflammatory cytokine TNF-α, which supports the extracellular Grp170 as a potential immunostimulatory “danger” molecule (104). Additionally, Hsp110 also induced mouse mammary carcinoma cells to elevate expression of IL-6, IL-12, and CD40 (102). A similar observation was made in mouse B16F10 melanoma cells treated with Grp170 (104).

The release of Hsp110 in subsets of human IECs into the intestinal lumen occurred as a consequence of epithelial renewal (61). The elevated levels of Hsp110 serve as an immunological “trigger,” resulting in expression of CD1d and subsequent activation of natural killer T (NKT) lymphocytes (61). We recently demonstrated that extracellular Grp170 can function as a chaperone for microbial DNA, CpG oligodeoxynucleotides (CpG-ODN), which facilities immune-mediated recognition and clearance of pathogens (66). Grp170 was highly efficient in binding CpG-ODN and potentiated the endocytosis of CpG-ODN by macrophages, which led to the enhanced activation of toll-like receptor 9 (TLR9) (66). It should also be noted that Grp170 is able to directly interact with TLR9 and this interaction increases during stimulation of TLR9 signaling. Furthermore, Grp170–CpG-ODN complex initiated innate immunity enhanced the protection of mice from challenge with *Listeria monocytogenes* compared to CpG-ODN alone (66). This finding uncovered a new feature of extracellular large HSPs in modulating the immune sensing of pathogen-associated molecular patterns (PAMPs). Together, the active cooperation between the extracellular chaperone and TLR9 may be essential for amplification of innate immunity and maintenance of the host homeostasis.

Exposure of mononuclear phagocytes or macrophages to hypoxia-induced upregulation of Grp170 expression, which was restricted to foam cells within atherosclerotic lesions (105). The biological function of Grp170 in macrophages may be linked to the survival or function of this inflammatory cell population that is known to contribute to the pathogenesis of atherosclerosis (106). Additionally, the expression of Grp170 was highly increased in alveolar macrophages and lung epithelial cells in mice upon lipopolysaccharide (LPS) challenge (107). Overexpression of the Grp170 was able to confer resistance to LPS-induced acute lung injury (107). However, the possible role of intracellular Grp170 in LPS-induced inflammatory response has not been examined. Recently, Giffin et al. reported that Grp170 was associated with viral interleukin-6 (vIL-6), a cytokine homolog encoded by human herpes virus 8 (52). This interaction increased the ability of vIL-6 to bind gp130, thereby potentiating vIL-6-induced JAK/STAT signaling, survival as well as migration of endothelial cells (52). This suggests that Grp170 can modulate vIL-6 function and promote pro-inflammatory signaling, angiogenesis, and cell proliferation.

## Large HSPs in Antigen Cross-Presentation

In light of exceptional protein-holding capacity and superior immunostimulatory activity of the large HSPs (Hsp110, Grp170), we have created recombinant heat shock vaccines by complexing clinically relevant tumor protein antigens to these large chaperones (74, 108–112). These reconstituted complexes exhibited potent antitumor activities by eliciting antigen-specific cytotoxic lymphocytes (CTL) responses prophylactically and therapeutically. Due to the nature of these soluble antigen-targeted protein vaccines, it is believed that antigens-chaperoned by large HSPs are introduced into an antigen cross-presenting pathway in professional antigen-presenting cells (APCs), e.g., DCs (9, 113, 114). Intracellularly processed antigenic peptides are then loaded onto major histocompatibility complex (MHC) class I molecules, followed by presentation to CD8^+^ T lymphocytes.

Despite the fact that these large HSPs efficiently facilitate a CD8^+^ T cell response to associated antigens, the mechanism underlying large HSP-enhanced antigen cross-presentation during interaction with DCs is not completely understood. The prolonged existence or stability of antigens is an important prerequisite for cross-presentation after internalization because antigen degradation by endo/lysosomal proteases can rapidly destroy putative antigenic epitopes (115). Hsp110 and Grp170 are significantly more efficient than other chaperones in stabilizing heat-denatured protein substrates (50, 72), which may help to protect antigenic epitopes from being degraded. In addition, these large HSPs may be able to influence intracellular trafficking of antigens to promote cross-presentation. Kutomi et al. showed that Grp170 shuttled the chaperoned peptide into the Rab5^+^EEA1^+^ static early endosomes and transferred the peptide onto the recycling MHC class I molecules (116).

Using a clinically relevant melanoma protein antigen (i.e., gp100), we recently investigated the trafficking pathway of Grp170–gp100 vaccine complex internalized (117). We showed that the Grp170 directed the antigen gp100 efficiently to the ER following uptake into DCs. Grp170-facilitated antigen processing and presentation was dependent on the endoplasmic reticulum-associated protein degradation (ERAD) pathway involving Sec61, which targets gp100 for proteasome-mediated degradation in the cytosol and subsequent integration into the conventional MHC class I restricted antigen-processing pathway. Grp170 can assist protein antigen to escape from lysosomal degradation and shuttle the antigen into the ER from the early endosomal compartment, possibly *via* a previously reported “ER–endosome fusion” process (118–120). Internalized Grp170 may be directly involved in the ERAD, because Grp170 in the complex enhanced the interaction of gp100 with several molecules known to participate in this pathway (e.g., Sec61α, VCP/97, CHIP, Grp78). It is conceivable that partially unfolded gp100 protein during the vaccine preparation serves as an ERAD target once accessing the ER. Since endogenous Grp170 also binds to Sec61α, it is possible that internalized Grp170 collaborates with other ER chaperones to guide antigen retrotranslocation. Interestingly, cytoplasmic chaperone Hsp110 was recently shown to regulate folding as well as quality control of cystic fibrosis transmembrane conductance regulator (CFTR) in the ER (62). It is of interest to determine whether endogenous Hsp110 is also involved in Grp170-enhanced antigen cross-presentation by DCs.

It has been established that antigen cross-presentation promoted by HSPs, including Hsp110 and Grp170, requires uptake of the HSP–antigen complexes by pattern recognition scavenger receptors (SR), such as CD91, LOX1, scavenger receptor A (SRA), and SREC (121–127). Although SRA serves as a binding structure on DCs for exogenous Hsp110 or Grp170, the loss of SRA does not seem to significantly alter the overall capacity of DCs to process and present Hsp110-associated antigen (i.e., gp100) (108, 127, 128). On the contrary, the lack of SRA increased the ability of DCs pulsed with large HSP–gp100 complex to stimulate gp100-specific naive T cells (108). Given that LOX-1 and SREC have been shown to potentiate Hsp70-mediated antigen cross-presentation (121, 125), we postulate that SRs are functionally distinct upon interactions with HSPs that carry antigens (129).

## Use of Large HSPs for Development of Cancer Vaccines

Numerous studies have documented the immunogenicity of tumor-derived HSPs, which has been attributed to the individually distinct array of antigenic peptides associated with these chaperone proteins (130–138). Our early study showed that vaccination of mice with Hsp110 or Grp170 purified from methylcholanthrene-induced fibrosarcoma resulted in a complete regression of the tumor (139). Moreover, tumor-derived Hsp110 or Grp170 appeared to elicit a more potent antitumor response on a molar basis than Hsp70 (139), which may be explained by their superior antigen chaperoning capability. Several reports suggest that the affinity with which the chaperone binds antigen determines its ability to induce a CTL response (140–142). Using a more aggressive and less immunogenic mouse B16 melanoma model, we demonstrated that tumor-derived Grp170 preparations delayed tumor progression and reduced pulmonary metastases (143).

HSP–peptide complexes derived from patient tumors represent an autologous or personalized vaccine. However, the yield of such a vaccine in the clinic is low for certain types of cancer due to requirement for patient specimen (144). To overcome this limitation, we developed a recombinant chaperone vaccine by reconstituting Hsp110/Grp170-tumor protein antigen complexes under heat shock conditions (72, 110, 112, 145). A “natural chaperone complex” between Hsp110 and the intracellular domain (ICD) of human epidermal growth factor receptor 2 protein (HER-2)/neu elicited both CD8^+^ and CD4^+^ T-cell responses against ICD. The Hsp110–ICD complex also significantly enhanced ICD-specific antibody responses relative to that seen with ICD alone (112). Subsequent studies showed that the Hsp110–ICD complex was able to inhibit the development of spontaneous mammary tumors in FVB-neu (FVBN202) transgenic mice (145). Consistent with this finding, immunization with melanoma antigen gp100 complexed with Hsp110 exhibited therapeutic efficacy against established B16 melanoma (72). Strikingly, Hsp110 was more effective than complete Freud’s adjuvant in inducing an antitumor immune response (72), which is likely to be due to the unique capability of the large HSPs in promoting antigen cross-presentation. The extensive preclinical studies by us and others have validated this recombinant chaperone vaccine approach that uses large HSPs to target tumor antigens to professional APCs (74, 108–111, 146–148), which provide strong scientific rationale for exploiting these large HSPs to develop synthetic and non-toxic vaccines for cancer immunotherapy.

The similar principle has been used to prepare a recombinant vaccine to induce an immune response to infection. McLaughlin et al. showed that recombinant Hsp110 can efficiently bind to complete viral antigens and enhance monocyte-stimulated proliferation of recall CD4^+^ T cells *in vitro*. However, the complexes failed to improve primary immune response *in vivo* (149). Therefore, more studies are necessary to understand immunoregulatory activity of the large HSPs in vaccine design directed against infectious diseases.

Using colon-26 (CT26) cancer cells stably transfected with Hsp110, we showed that Hsp110 overexpression markedly enhanced the immunogenicity of the tumor *in vivo*. Immunization of mice with irradiated CT26-Hsp110 cells caused growth inhibition of unmodified CT26 tumor, associated with increased frequency of tumor-specific T cells (150). Similarly, engineering of TRAMP-C2 mouse prostate cancer cells to secrete Grp170 profoundly enhanced tumor immunogenicity, indicated by increased levels of tumor-infiltrating CD8^+^ T cells, enhanced cytolytic activity, and improved control of distant tumors (104), suggesting that the induction or manipulation of large HSPs for secretion may help break immune tolerance to cancer cells. We also found that the secreted Grp170 chaperoned full length tumor protein antigens, which can be potentially captured by DCs in the tumor microenvironment (151). Considering abundant tumor antigens present in cancer cells, we investigated the feasibility of intratumoral delivery of Grp170 using an adenovirus to promote antitumor immunity. We showed that the adenovirus encoding a secretable form of Grp170 elicited a tumor-reactive CTL response (152). Furthermore, this Grp170-expressing adenovirus combined with an adenovirus encoding melanoma differentiation-associated gene-7/interleukin-24 (*mda*-7/IL-24), a cancer-specific, apoptosis-inducing gene (153), led to a synergistic systemic antitumor effect as shown by improved control of both treated and untreated prostate cancers (152).

The significance of pathogen-sensing TLR signaling in enhancing antigen presentation by specialized APCs (e.g., DCs) and in bridging innate and adaptive immune responses has been well established (154). Incorporating pathogen-associated molecules or TLR agonists into therapeutic vaccines can potentially augment immune activation. Recently, we engineered a chimeric chaperone by fusing Grp170 with a defined NF-κB-activating domain of the TLR5 agonist flagellin. This chimeric molecule, termed Flagrp170, combined action of the large HSP in facilitating antigen cross-presentation and a microbial immunostimulatory signal for functional activation of DCs (155). Intratumoral administration of an adenovirus expressing Flagrp170 restored systemic antitumor immunity against B16 melanoma and distant lung metastases compared to either unmodified Grp170 or Flagellin. The therapeutic potency of Flagrp170 was also confirmed in mouse models of prostate cancer and colon cancer. The mechanistic studies showed that Flagrp170-provoked activation of tumor-reactive T cells required CD11c^+^ DCs and CD8^+^ DCs. Although research is needed to better understand the molecular and cellular bases of immunoregulation by this chimeric chaperone in the immunosuppressive tumor environment, our results support the use of this new generation chaperone molecule for future design of optimized vaccines to achieve improved treatment outcomes. In a separate study, Chen et al. reported that Grp170-HPV16 E7_49–57_ peptide complex plus the TLR3 agonist polyinosinic-polycytidylic acid or poly(I:C), a synthetic analog of double-stranded RNA and a molecular pattern associated with viral infection, induced synergistically an immune response to cervical cancer in mice (156). Therefore, strategic inclusion of a microbial component in large HSP-based vaccine regimen will further strengthen its immunostimulatory capacity in driving an effective CTL response for tumor eradication.

The levels of HSPs, including large HSPs, are generally elevated in various human tumors possibly due to the increased requirement for chaperone molecules to stabilize the mutated or oncoproteins in cancer cells (41, 157). A recent study established a direct correlation between Hsp110 expression and lymphoma aggressiveness (63). Treatment of human B-NHL cell lines with an anti-Hsp110 antibody had no direct effect on cell cycle or apoptosis, but significantly reduced the tumor burden in xenotransplanted immunodeficient mice (63). Hsp110 was reported as a prognostic biomarker for the poor survival of patients with breast cancer and melanoma (41). Similarly, both the protein and mRNA levels of Grp170 were significantly upregulated in several cancer cell lines compared to their normal counterparts (158). Induced overexpression of Grp170 inhibited the senescence and apoptosis of human breast cancer cells, not normal cells (158). Considering the potential involvement of these large HSPs in oncogenesis, Hsp110 or Grp170 itself may represent a novel tumor-associated antigen for immunotargeting. Indeed, vaccination with Hsp110 cDNA resulted in growth inhibition of colorectal CT26 and melanoma B16 tumors, which was associated with stimulation of both Hsp110-specific CD4^+^ and CD8^+^ T cells (159). Although our recombinant chaperone vaccine does not appear to induce a significant cellular or humoral response to the Hsp110 (112), such a response may be ideal for those cancers with high expression of the large HSPs.

## Large HSPs and Inflammatory Diseases

Inflammatory disease is a term that collectively describes a group of apparently unrelated conditions that have common inflammatory pathways leading to inflammation, which may result in various organ damages (160, 161). Although each of these diseases has distinctive epidemiology and pathophysiology, the dysregulated inflammatory response is believed to be pivotal to the disease pathogenesis. In addition to conventional inflammatory disorders, such as inflammatory bowel disease (IBD), diabetes, rheumatoid arthritis (RA), and multiple sclerosis (MS), some other diseases, such as idiopathic pulmonary fibrosis (IPF), myocardial infarction (MI), were recently shown to involve inflammatory responses as well. Despite that HSPs have been implicated in inflammatory and immune-mediated diseases over the past few decades (162–166), the roles of the large HSPs in disease pathogenesis, especially in immune-related processes, remain less investigated. Understanding of molecular actions of Hsp110 and Grp170 in classical inflammatory diseases and those that involve an inflammatory process may provide new insights into disease mechanisms and lead to new strategies for prevention and therapy of these diseases.

An early study by Colgan et al. showed that Hsp110 released from IECs contributes to CD1d surface expression in a novel autocrine pathway, suggesting that Hsp110 regulation of CD1d represents the “physiologic inflammation” in mucosal tissue sites (61). Since CD1d presents self and microbial lipid antigens to NKT cells, it is possible that the Hsp110–CD1d axis might contribute to the pathogenesis of inflammatory diseases, e.g., IBD. Surprisingly, a recent study from this group reported that bone-marrow-derived CD1d signals induced NKT cell-mediated intestinal inflammation; however, engagement of epithelial CD1d elicits protective effects through the STAT3-dependent regulation of IL-10, Hsp110, and CD1d itself (167), which highlights a role of Hsp110 in IEC-dependent maintenance of gut homeostasis. In line with these observations, pharmacological stimulation of Hsp110 expression may be exploited to prevent colorectal inflammation and favorably affect the progression of IBD. Indeed, human HSP A4 (HspA4, also called Apg2), a member of the Hsp110 family, is inducible by chronic inflammation (164, 168). A recent study showed that increased HspA4 inhibited apoptosis of inflammatory cells, thereby augmenting immune response in the gut through the upregulation of Bcl-2 and IL-17 expression, which led to treatment resistance in IBD (164). This result also suggests that HspA4 may be used as a potential biomarker for refractory IBD. Interestingly, Zebrafish HspA4 was also upregulated in the intestinal epithelium within the gut under inflammatory stress conditions (169). Using HspA4-deficient mice and human tissue samples, Sakurai et al. showed that the expression of HspA4 was inversely correlated to gastric ulcer healing induced by endoscopic submucosal resection (170). Further studies revealed that HspA4 downregulated the expression of stromal cell-derived factor 1 (SDF-1, also known as CXCL12), which signals through its cognate receptor CXC chemokine receptor 4 (CXCR4). The resultant inhibition of cell migration delayed gastric ulcer healing (170).

Obesity and associated insulin resistance predispose individuals to develop chronic metabolic diseases, such as type 2 diabetes. Emerging evidence supports an important role for Grp170 in insulin release (171–173) or insulin resistance in diabetes (174, 175). Kobayashi et al. showed that Grp170 was highly expressed on pancreatic beta cells and decrease in serum glucose concentration by fasting strongly suppressed Grp170 expression in the pancreas concomitant with decreased insulin level in the serum (172). Interestingly, titer of autoantibodies against Grp170 was elevated during high-fat diet feeding (171). A significant increase in autoantibodies to Grp170 was also observed in patients with type I diabetes (176). However, the pathophysiological effect of these autoantibodies to Grp170 in diabetes remains unclear.

Involvement of Grp170 in improving insulin sensitivity of the skeletal muscle and liver was established in type 2 diabetes (175). Overexpression of Grp170 was shown to delay the onset of disease and improve insulin sensitivity, subsequently ameliorated glucose tolerance in diabetic animals. Conversely, reduction of Grp170 in the liver facilitated the disease progress and decreased insulin sensitivity (174). Mechanistic studies suggested that the improved insulin sensitivity by Grp170 was executed though attenuation of oxidative stress and augmentation of insulin signaling in the skeletal muscle and liver (174). Recently, Deng et al. showed that Grp170 expression on pancreatic beta cells decreased gradually during the pathogenesis of acute necrotizing pancreatitis (177). Dekki et al. reported that transthyretin (TTR), which promotes insulin release and protects against pancreatic beta cell death, was associated directly with Grp78, Grp94, and Grp170 in pancreatic beta cells (178). Treatment of beta cells with physiological concentrations of TTR triggered a pronounced increase in intracellular calcium concentration (179). The TTR-induced change in calcium levels was abolished when cells were treated with an antibody against Grp78 (178). Although Grp170 was not directly studied in this model, it is likely that Grp170 along with other chaperone molecules in these multimeric complexes collaboratively regulate the internalization or activity of TTR, thereby affecting insulin release. Several studies have shown that intracellular Grp170 was involved in regulation of calcium signaling or calcium homeostasis (58, 180, 181). However, it remains to be determined if such a pathway alters the function of pancreatic beta cells and inflammatory processes associated with diabetes.

Idiopathic pulmonary fibrosis is a progressive chronic disease associated with inflammatory responses, fibrosis, and lung dysfunction. Treatment of mice with bleomycin-induced lung damage with concurrently enhanced expression of Grp170 in the lung (182). Despite a modest exacerbation of inflammatory responses in Grp170^+/−^ mice, these animals showed significantly ameliorated pulmonary fibrosis, alteration of respiratory dysfunction compared to wild-type counterparts. Although Grp170 appeared to be a protectant against bleomycin-induced lung injury, it promoted lung fibrosis by increasing levels of TGF-β1 and myofibroblasts (182).

Myocardial infarction (i.e., heart attack) is the irreversible myocardial cell damage or death, which occurs during prolonged ischemia caused by blockage of a coronary artery. Grp170 overexpression significantly reduced the hypoxia/reoxygenation-induced cardiomyocyte death by inhibiting activation of capase-3 and release of mitochondrial cytochrome C. This protective effect of Grp170 also inhibited injury caused by myocardial ischemia–reperfusion *in vivo* (181). In addition, increased levels of Grp170 and Grp170-derived peptide fragments was shown in the plasma of patients after MI, which was associated with enhanced ER or hypoxic stress and suggested to be prognostic marker that predicts a poor outcome (183). Accumulating evidence indicates that MI-triggered inflammatory response is involved in injury, repair, and remodeling of the infarcted heart (184, 185). Recent study showed that stimulation of inflammasomes induced the caspase activation associated with maturation and secretion of biologically active IL-1β, which can cause additional loss of functional myocardium and heart failure in mouse model of MI (186). While intracellular Hsp90 has been reported to regulate activation of inflammasome (187), the potential effect of intracellular or extracellular Grp170 on inflammasome activity as well as in the pathophysiology of MI remain to be examined. Elucidating the inflammatory pathways and their contributions to the pathogenesis of MI may lead to novel therapies for preventing post-infarction heart failure.

**Figure 1:**
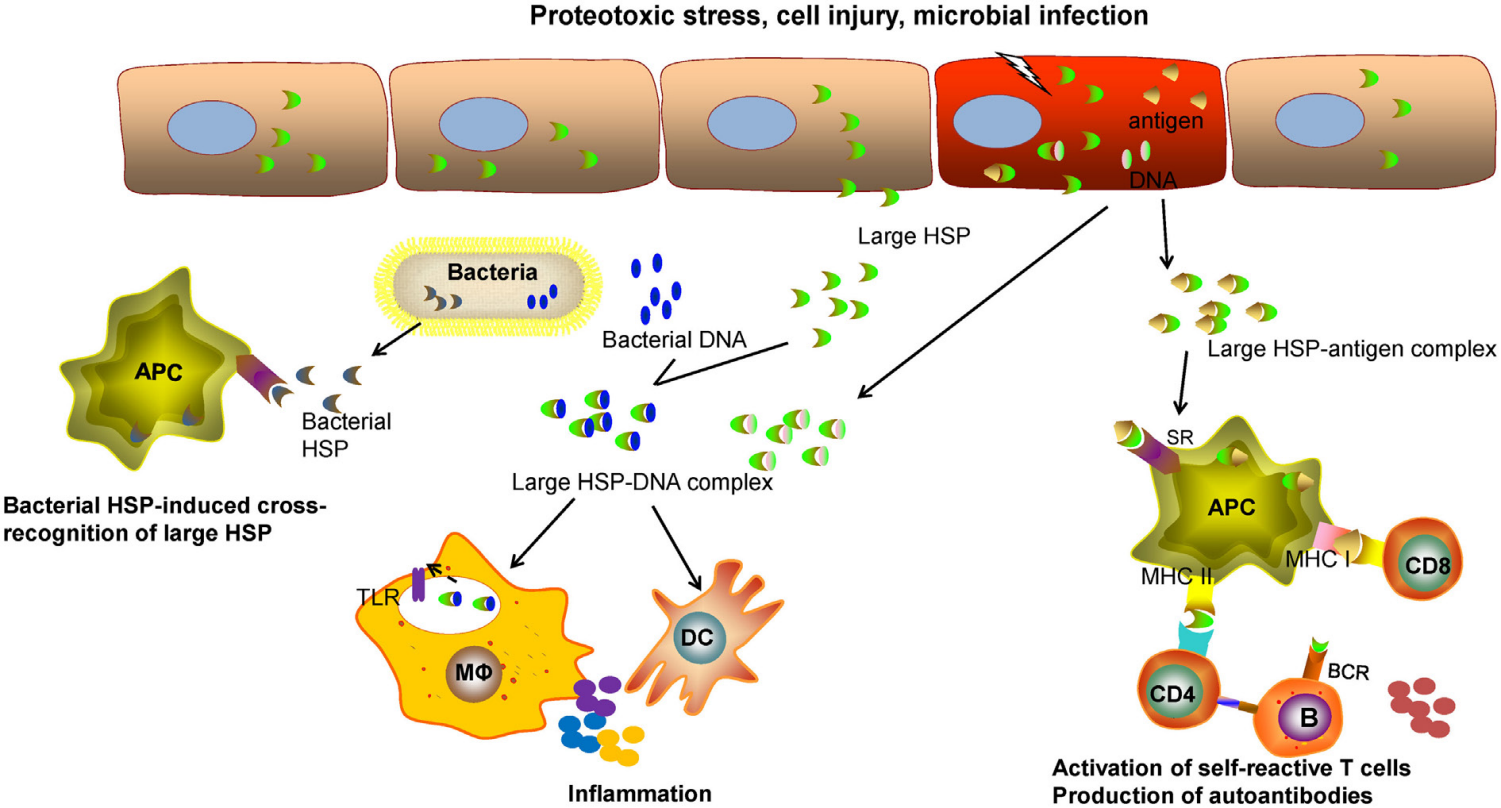
**The potential role of large HSPs in inflammatory or immune-related diseases**. Intracellular HSPs typically function as molecular chaperones to promote proteostasis, counteract cellular stresses, and improve cell survival. The extracellular large HSPs, released from the host cells, caused by proteotoxic stresses, cell injury, or microbial infection, may be involved in regulation of immune pathways that contribute to immune pathologies. The large HSPs complexed with self-antigens can be captured by professional antigen-presenting cells (APCs) through scavenger receptor (SR)-mediated endocytosis. Large HSP-based chaperoning enhances the cross-presentation of associated antigens and activation of self-reactive CD8^+^ T cells. The processing of these HSP–antigen complexes may result in activation of CD4^+^ T cells and production of autoantibodies by B lymphocytes. It is also likely that a cellular or humoral response directed to the large HSP itself can be induced in certain pathological conditions. The released large HSPs from injured cells, if complexed with self-DNAs or with microbial DNAs in the case of infection, can provoke and amplify inflammation or an innate immune response triggered by toll-like receptors (TLRs) present on sentinel cells, such as macrophages (Mφ) or dendritic cells (DCs). Due to the conserved sequence, pathogen-derived microbial HSPs from (e.g., bacteria) may induce an immune response that cross-reacts with self HSPs during chronic infection.

Although large HSPs, like other chaperone molecules, function to primarily promote and restore cellular or tissue homeostasis, they may be involved in immune pathology through several mechanisms given their potential immunoregulatory effects (Figure 1). Of note, extracellular Grp170 secreted from intact cells or released from injured cells facilitate the delivery of Grp170–antigen complex to DCs *via* interaction with surface SRs, thereby enhancing the cross-presentation of the HSP-bound antigens for T cell activation (9, 113, 114, 117). In addition to functioning as “danger” molecules that alert the immune system of tissue damage (102, 103), the extracellular Grp170 has a capacity to amplify the inflammatory response triggered by microbial signal (66) and possibly endogenous damage-associated molecular patterns (DAMPs) as well. Indeed, host-derived DAMPs, such as RNA (188) and DNA (189, 190), can also be recognized by TLRs or other pattern recognition receptors that provokes sterile inflammation or inflammatory diseases (191–194). Compared with healthy individuals, serum from patients with autoimmune diseases, patients with trauma, and children with septic shock shows high concentrations of Hsp70 (59), even though Hsp110 or Grp170 was not examined in this study.

Cross-recognition of the self and the mycobacterial HSPs by T cells suggests that HSPs may act as a link between infection and autoimmunity (195). Although the controversy remains, the molecular mimicry caused by structural similarity between microbial HSPs and mammalian HSPs may lead to autoimmunity to self HSPs following bacterial infection (196–198). Consistent with this idea, Hsp110 itself can be immunogenic in certain contexts, such as DNA vaccination against Hsp110 that resulted in priming of T cells reactive with Hsp110 on tumor cells *in vivo* (159). Increased levels of anti-Hsp110 antibodies and enhanced expression of Hsp110 have been observed in mice with experimental autoimmune encephalomyelitis (EAE) as well as patients with MS (199), which support a potential role of Hsp110 in inflammatory autoimmune pathology.

Although therapeutic inhibition of Hsp90 has been evaluated in the context of cancer treatment (200), growing evidence indicates that Hsp90 inhibitors also provide benefits for treatment of inflammatory disorders. Inhibition of Hsp90 using a small molecular inhibitor prevented LPS-induced NF-κB activation and nitric oxide production, and attenuated the inflammatory response in EAE (201). Inhibition of Hsp90 reduced the activation of the transcription factors STATs and NF-κB by pro-inflammatory cytokines in atherosclerotic mice (202). These results suggest involvement of Hsp90 and potentially other HSPs (e.g., Hsp110, Grp170) in an inflammatory response possibly through modulating key regulators of immune signaling pathways such as JAK/STATs, TLR4, and NF-κB. We recently showed that UPR response enhanced the production of inflammatory cytokines (e.g., IFN-β) by DCs stimulated by poly(I:C), which involved both TLR3 and melanoma differentiation-associated gene-5. This enhanced inflammatory response was associated with increased activation of NF-κB and IRF3 signaling as well as the splicing of X-box-binding protein-1 (XBP-1), a transcription factor known to regulate ER chaperone genes such as Grp170 (203). It is not clear if the induction of intracellular Grp170 and/or secretion of Grp170 contribute to UPR-amplified inflammatory response in this context. Moreover, Grp170 is known to bind with immunoglobulin in B cells (51) and was suggested to functionally compensate for Grp94/Gp96 to facilitate the assembly of immunoglobulin (204), implicating a potential role of Grp170 in B cell functions. Strikingly, lupus-like autoimmune disorder and systemic inflammation are induced in Grp94/Gp96 transgenic mice, in which Grp94/Gp96 was engineered for cell surface expression (205). Thus, development of pharmacological inhibitors selectively targeting Hsp110 or Grp170 may provide new opportunities to treat certain inflammatory or immune-related diseases (Table 1).

**Table 1:** **The activity of large heat shock proteins in multiple diseases**.

	Pathology	Activity	Reference
Hsp110	Traumatic brain injury	Reduce injury at impact site	(22)
	Amyotrophic lateral sclerosis	Prevent the neuron toxicity mediated by mutant SOD1 protein	(90)
	Alzheimer’s disease	Maintain a proper folding environment for phosphorylation and dephosphorylation of tau and APP processing	(93)
	Cancer	Upregulate β-catenin and transcription of Wnt-targeted genes; suppress cancer cell apoptosis	(41, 97)
	Inflammatory bowel disease	Impair CD1d signal induced NKT cell-mediated intestinal inflammation; enhance treatment resistance by upregulating Bcl-2 and IL-17	(164, 167)
	Gastric ulcer healing	Delay wound healing by suppressing the expression of stromal cell-derived factor 1	(170)
	Multiple sclerosis	Increased levels of anti-Hsp110 antibodies	(199)
Grp170	Alzheimer’s disease	Maintain neuroprotective functions of sAPP alpha	(92)
	Acute lung injury	Protect alveolar cells after LPS exposure	(107)
	Obesity and diabetes	Promote insulin release and enhance the insulin Sensitivity; increased autoantibodies to Grp170	(171, 173–176)
	Myocardial infarction	Inhibit activation of capase-3 and release of mitochondrial cytochrome C	(181)
	Idiopathic pulmonary fibrosis	Protect against bleomycin-induced lung injury; promote lung fibrosis by elevating levels of TGF-β1	(182)

## Conclusion

Like other chaperone molecules involve in intracellular protein quality control, Hsp110 and Grp170 play essential roles for maintaining and restoring protein homeostasis under physiological and stress conditions. The upregulation of these large HSPs as part of stress response (e.g., heat shock response, UPR) generally provide a cytoprotective effect *via* assistance with protein refolding. Therefore, their manipulation may be strategically used to slow progress of certain diseases associated with protein misfolding or aggregation (e.g., neurodegeneration) or treat cancers that highly express Hsp110 or Grp170 to maintain oncogenic signaling for sustained proliferation and survivals. Due to their superior capacity to hold and target protein antigens for DC-mediated cross-presentation, these large HSPs have been used to develop antigen-targeted cancer vaccines and to enhance the immunogenicity of cancer cells. The efficient generation of an antitumor immune response by large HSP-based immunotherapy in animal models of several cancers has led to an ongoing phase I clinical trial in patients with advanced melanoma. Despite that these large HSPs are typically considered to be homeostatic factors in maintaining cellular function and tissue integrity, it is not surprising that they are involved in certain immunopathologies (e.g., inflammatory or immune-related diseases) given the well-documented immunoregulatory activities of surface or extracellular HSPs including the large HSPs at the interface of innate and adaptive immunity. A better understanding of multifunctional roles of Hsp110 or Grp170, defined by their expression compartmentalization as well as the nature, magnitude, or duration of stress signals in different contexts, will offer important insight into their pathogenic relevancies and assist in the design of new potential therapies for treatment of inflammatory disorders (e.g., IBD, MS).

## Author Contributions

All authors listed, have made substantial, direct, and intellectual contribution to the work, and approved it for publication.

## Conflict of Interest Statement

The authors declare that the research was conducted in the absence of any commercial or financial relationships that could be construed as a potential conflict of interest.
